# Identification of Vibration Modes and Wave Propagation of Operational Rails by Multipoint Hammering and Reciprocity Theorem

**DOI:** 10.3390/ma15030811

**Published:** 2022-01-21

**Authors:** Kodai Matsuoka, Kazuhiro Kajihara, Hirofumi Tanaka

**Affiliations:** 1 Railway Technical Research Institute, Railway Dynamics Division, 2-8-38, Hikari-cho, Kokubunji-shi, Tokyo 185-8540, Japan; 2 Railway Technical Research Institute, Track Technology Division, 2-8-38, Hikari-cho, Kokubunji-shi, Tokyo 185-8540, Japan; kajihara.kazuhiro.70@rtri.or.jp (K.K.); tanaka.hirofumi.96@rtri.or.jp (H.T.)

**Keywords:** vibration mode, rail damping, wave propagation, field test, rail corrugation

## Abstract

Vertical bending vibration modes and rail wave propagation, including the damping characteristics, are the factors that cause rail corrugation. However, the ability to identify actual railways has been limited because of the huge number of sensors required for field tests. In this study, a novel and field-applicable method for identifying rail vibration modes and wave propagation characteristics is developed by multipoint hammering and the reciprocity theorem instead of multipoint measuring. Additionally, the proposed method is applied to an actual rail with a direct fastening track system on a bridge that has corrugation with a wavelength of approximately 0.04 m. As a result, the wavelength (wavenumber)-, group velocity-, and distance damping (attenuation) frequency relationship of the wave propagation is clarified in addition to the rail frequencies and mode shapes up to approximately 1500 Hz, including the pinned-pinned mode. Finally, the identified wavelength-frequency relationships and the measured rail irregularity can empirically demonstrate that the generated corrugation on the rail is produced by wave interference on the two axles in the bogie.

## 1. Introduction

Vibration and wave propagation, including the damping of the rails that support the traveling axles, affect the wheel–rail interaction force. As a result, these can cause real problems on railways, such as rolling noise and rail corrugation [1,2,3,4,5,6,7,8]. Different generating mechanisms have been proposed for rail corrugation, depending on the corrugation’s wavelength [3]. Manabe [4] established that rail vertical bending modes and vertical bending wave propagation are related to the growth mechanism of rail corrugation, with a medium wavelength of approximately 0.04 m. It is typical of this type of rail corrugation that it can occur in straight lines as well. The objective of this study is to develop a methodology for identifying the vertical bending modes and vertical bending wave propagation characteristics of rails related to the generation of such rail corrugation by field tests and to demonstrate its effectiveness in an actual field. In light of this, this study focuses on the rail’s vibration mode, and wave propagation characteristics in the frequency range of approximately 500–1200 Hz, which is indicated to be caused by this type of wave interference [4], are targeted.

Many researchers have thus far contributed to the clarification of rail vibration modes. In particular, the relationship between short-pitch rail corrugation and pinned-pinned resonance at about 1 kHz has been reported [2,3,4]. In addition, a wavelength fixing mechanism for rail corrugation due to the interference of rail vertical bending mode waves has been proposed [3,4]. Most of these findings are based on theoretical analysis or numerical simulation. Many studies, such as rail wave propagation, are extensive. Many models have been developed to reproduce the rail dynamic behavior, such as beam or plate model analysis [5,9,10,11,12], semi-analysis or the 2.5D FE model (SAFE) [13,14,15,16], and the 3D FE model [17,18,19,20,21]. These provide basic information for understanding the wave number and frequency dispersion relations and the wave propagation characteristics such as phase velocity and group velocity. In addition, the dispersion curve, phase, and group velocity of rail waves for rail inspection have been examined in many studies [15,16,22].

However, most of these studies are based on numerical analysis. As a result, there are very few examples of rail modal characteristics and wave propagation characteristics identified in experiments and field tests [14,20,21]. Cettour et al. [14] proposed a method using wavelet transform to analyze the characteristics of waves propagating in the rail. In addition, the group velocity dispersion curve of the vertical, lateral, and longitudinal rail waves at 1 to 7 kHz was estimated by applying it to a relatively short rail in the laboratory [14]. In their study, a method using one-point excitation and measurement (accelerometer) was proposed, but problems with wave reflection at the rail ends remained. Zhang et al. [18,19] has proposed the synchronous multiple acceleration wavelet (SMAW) approach, using multiple accelerometers as a method to solve the problem. The effectiveness of the method was also verified on relatively short laboratory rails. In addition, with the help of numerical analysis, the main vibration mode shapes and wavenumber-frequency dispersion relation were identified. Attempts have been made to apply it to actual rails in the field, but no wavenumber–frequency relationship has been obtained. Despite these significant contributions, the following challenges exist in identifying rail vibration modes and wave propagation characteristics.

- In rail mode identification, the number of accelerometers (spatial resolution) is insufficient, especially when identifying rail mode shapes. Even in the latest literature [20,21] using a relatively large number of sensors, the mode shape of the pinned-pinned mode has not been completely experimentally identified.
- The verification is limited to short-length rails in the laboratory, and the influence on experimental conditions such as the free ends cannot be denied. In particular, the identification of the wavelength-frequency relationship of actual rails is deeply related to the rail corrugation in [4], but they have remained unknown until now.

In this study, the authors propose a new approach using multipoint hammering and the reciprocity theorem as a method of solving the constraint on the number of sensors. The concept of the proposed method is to substitute the multipoint sensor measurement with a multipoint hammering excitation through the reciprocity theorem, which exchanges the measured and excited points. In modal identification, the reciprocity of the frequency response function (FRF) holds, and, theoretically, modal identification based on multipoint measurement in [20,21] can be replaced with multipoint hammering [23,24,25]. In addition, the SMAW measurement [21] for estimating the wave propagation characteristics requires the reciprocity of wave propagation. As for this, Schmerr and Song [26] and Kubrusly et al. [27] show that the reciprocity of the surface integration consisting of the inner product of the surface force and the displacement of the dynamic elastic body is established. Therefore, the wave propagation characteristics can also be identified by multipoint hammering in principle.

Unlike in multipoint synchronous measurement, the magnitude and frequency characteristics of the input load fluctuate with each excitation in multipoint hammering. Therefore, the wavelet transforms of the measurement points such as SMAW measurement [21] cause an error in the identification result of distance damping (attenuation) due to these excitation characteristics. In this study, the excitation force obtained at the same time as the acceleration by the hammer is also wavelet-transformed, and the wavelet power spectrum (WPS) of the acceleration is standardized by the WPS of the hammering force at each central frequency of the wavelet. This can suppress fluctuations in hammering characteristics. This makes it possible to identify rail modal characteristics and wave propagation characteristics by multipoint hammering and the reciprocity theorem. In this study, the proposed method, called the SMEW (synchronous multiple excitation wavelet) approach, is based on the multipoint excitation and reciprocity theorem.

The proposed method has the following advantages over multipoint measurement: with only one sensor is required for the proposed method, and with an extremely simple measurement system configuration that includes one hammer, it is possible to obtain information equivalent to multipoint synchronous measurement with a huge number of measurement points. Therefore, field tests such as high-order (short wavelength) vibration mode shapes, wavelength-frequency dispersion relations, and unsteady wave distance attenuation can now be empirically identified by field tests; they were difficult to verify by field tests due to restrictions on the number of sensors. In this way, it is possible to analyze the effects based on the conditions unique to each site and each rail in addition to the general knowledge obtained in theory and laboratories in the investigation of the causes of rail corrugation and the growth process. In addition, it is possible to verify the effect of countermeasures from the viewpoint of the actual mode shapes and wave propagation. In fact, in this study, the proposed method is applied to an actual corrugated rail with a direct fastening track system on a bridge over an operational railway. As a result, the cause of rail corrugation is clarified from the identified wave propagation characteristics in this study.

## 2. Methods of Measurements and Analysis

### 2.1. Outline of the Proposed Method

**Figure 1:**
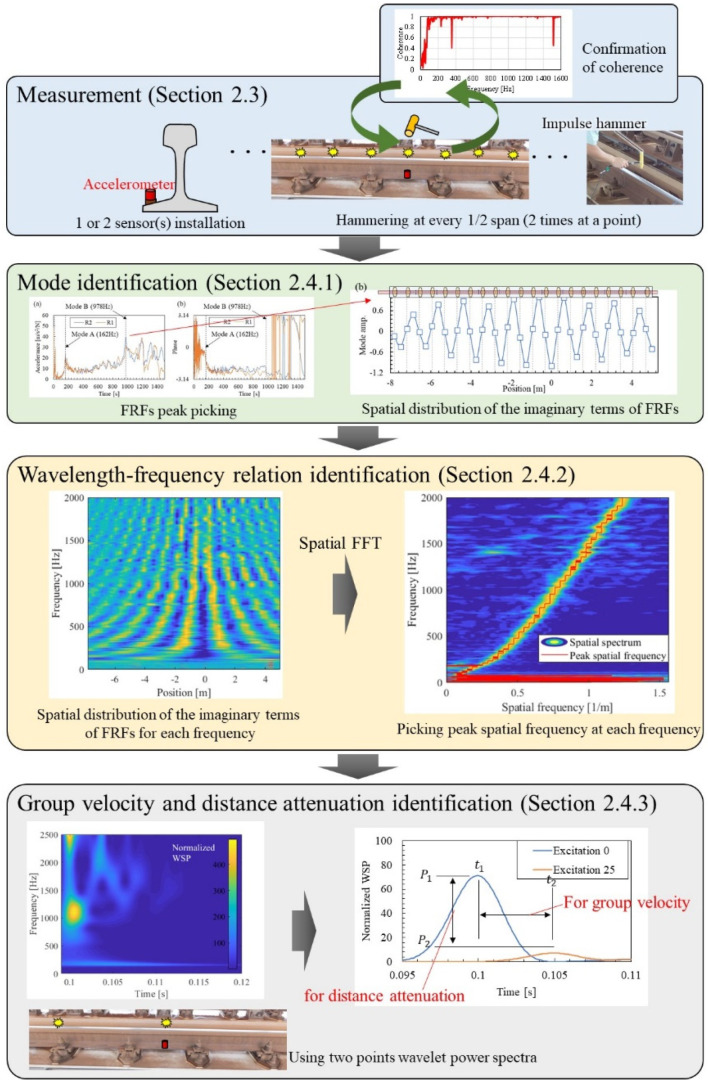
Flow of the proposed measurement and identification method.

Figure 1 shows the outline of the proposed measurement and identification method. The proposed method consists of four steps: measurement, mode identification, wavelength-frequency relation identification, group velocity, and distance attenuation identification. This allows the modal and wave propagation characteristics of the rail to be identified completely by measured data and driven without the use of any numerical simulations such as a past contribution [21]. The full-empirically identification of the characteristics of rails that require many measurement points, such as a pinned–pinned mode shape and the wavelength-frequency relation, is a unique contribution of this research that has not been achieved in past studies.

High-density (each 1/2 span) multipoint hammering achieved by the reciprocity theorem significantly reduces the working time required for field testing. In this study, a total of 41 hammerings were performed. This work was conducted locally in about an hour. The time required for the field test is about 2 h, including the installation and detaching time of the two accelerometers. On the other hand, with a 41-point accelerometer, these installations and wiring would take more than a day. It is difficult to perform this on the operational rails through which trains pass. Therefore, the proposed method can provide important information about operational rails that was not previously available in field tests.

Each part constituting the proposed method, as shown in Figure 1, is explained in detail in the sections shown in the figure.

### 2.2. Tested Rail

**Figure 2:**
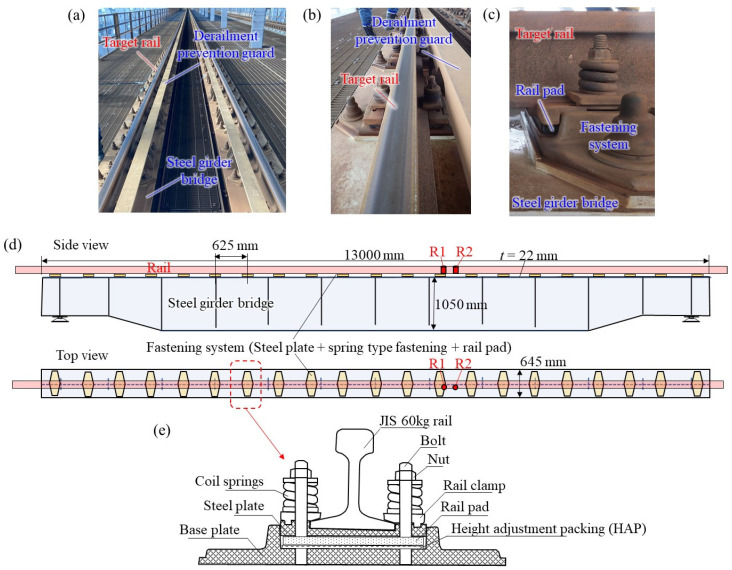
Tested rail: (**a**) rails, (**b**) tested rail (enlarged), (**c**) fastening system, (**d**) tested rail and supporting bridge drawings, and (**e**) fastening system drawing.

**Figure 3:**
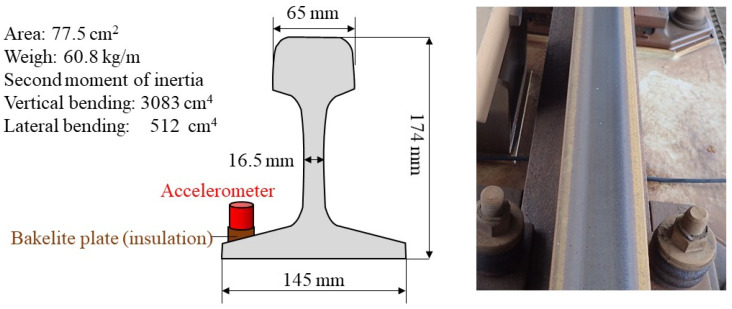
Test rail specifications and occurrence of rail surface irregularity (corrugation) and sensor position.

A rail on the actual line targeted in this study is shown in Figure 2. The rail is a JIS 60 kg rail (height of 174 mm) [4] that is standardly used in Japanese high-speed railways, as shown in Figure 3. This rail is laid by a direct fastening track system on the steel bridge via a fastening system. Therefore, there are no effects such as sleepers and ballast. Since the rail is a long rail, it is assumed that it is close to an infinite length beam where the influence of the rail end is small. The I-girder steel railway bridge is sufficiently stiffer than the rail, and it is considered that the vibrational coupling with the rail can be ignored. The fastening interval is about 625 mm. The rail is laid on the steel base via rail pads and height adjustment packing (HAP) and fixed by a spring-loaded fastening system. The static elastic stiffness of the rail pad, including HAP, is 39.5 MN/m, as evaluated by a separate laboratory test. The rail is in a straight section. In addition, the rail has been in place for almost 30 years. Corrugation with a wavelength of approximately 40 mm was observed on the rail under these conditions, as shown in Figure 3. However, the irregularity (depth) is less than 0.05 mm, which is very small.

### 2.3. Measurement Methods

**Figure 4:**
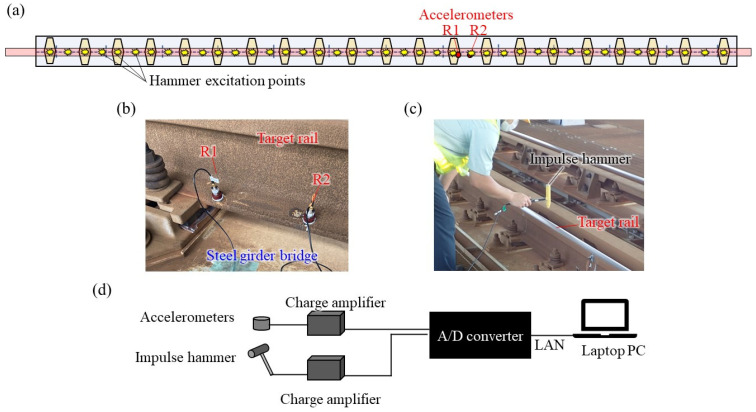
Measuring method: (**a**) hammering and measurement points’ arrangement, (**b**) accelerometer installation, (**c**) rail hammering, (**d**) configuration of measuring equipment.

The measuring method performed in this study is shown in Figure 4. The rail mode shapes, including the pinned–pinned mode and the wavelength-frequency relation in wave propagation, are identified by performing a high-density hammering point arrangement instead of a high-density sensor arrangement. Therefore, there are only two accelerometers, shown as R1 and R2, in Figure 4a,b. Originally, it could be identified with only one sensor, not considering the influence of the fastening. Taking this into consideration, two points were set at the mid-span of the rail’s fastening span and near the fastening system. Accelerometers were installed at the bottom of the rail, as shown in Figure 3. It has been reported that at high frequencies, the deformation of the rail cross-section is added to the deformation in the vibration and wave propagation modes, although the effect of the cross-section deformation is negligible below approximately 1200 Hz [21]. In addition, since trains travel through this rail daily, the sensors were installed at the bottom of the rail, avoiding the rail head. Hammerings were conducted at train passing intervals for the rail head at the 41 locations shown in Figure 4a, above the fastening and mid-span of the fastenings. The hammerings were performed in the vertical direction. The hammering state is shown in Figure 4c. The exciting force and acceleration signals observed by hammering were recorded on a laptop PC at 10 kHz via a charge amplifier and A/D converter. Table 1 shows a list of equipment used for the measurement.

**Figure 5:**
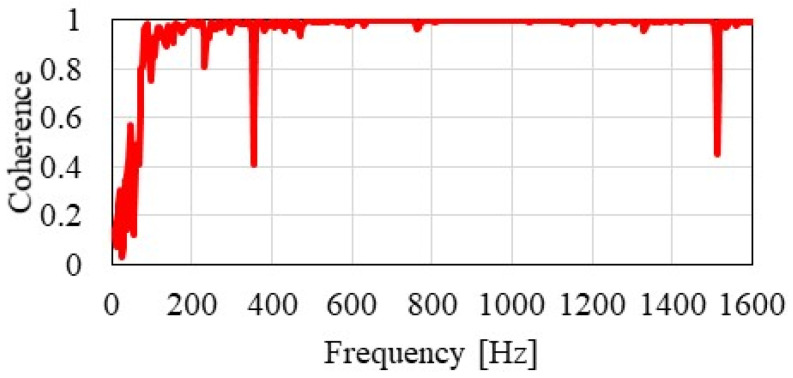
Measured coherence between acceleration and excitation force responses.

Coherence between acceleration and excitation force was calculated after each hammering, and it was confirmed that sufficient coherence could be obtained up to 1500 Hz. Figure 5 shows an example of calculated coherence. Hammering was performed up to 2–4 times per excitation point, and two pieces of high-coherence data were recorded. Only one piece of hammering data per point is used in the analysis below.

### 2.4. Identification Methods of Vibration Modes and Wave Propagation

#### 2.4.1. Modal Identification Method by Multipoint Vibration and Reciprocity Theorem

FRF is a basic method used for the mode identification of many structures [20,21,28,29]. The exciting force at the arbitrary point m and the acceleration at the arbitrary point l are both measured in this section. At this time, the accelerance $G_{lm}\left(\omega \right)$, which is one of the FRFs, is expressed by the following Equation (1).

(1) $$G_{lm}\left(\omega \right)\;=\;\frac{A_{l}\left(\omega \right)}{F_{m}\left(\omega \right)}=\sum_{r=1}^{N}\frac{-\omega^{2}\cdot \Phi_{lr}\Phi_{mr}}{\omega_{r}^{2}-\omega^{2}+i\cdot 2\zeta_{r}\omega_{r}\omega }.$$

Here, $A_{l}\left(\omega \right)$ is the Fourier transform of the acceleration response at the measurement point l, $F_{m}\left(\omega \right)$ is the Fourier transform of the vibration force at the excitation point m, ω is the circular frequency (ω = 2πf, f is frequency), $\omega_{r}$ and $\zeta_{r}$ are r-th frequencies and modal damping ratios, $\Phi_{lr}$ and $\Phi_{mr}$ are the r-th mode shapes at points l and m, and i is an imaginary unit. The frequency of the vibration modes is identified by peak picking because FRFs show peaks at the frequency $\omega_{r}$ from Equation (1).

Here, it can be assumed that the following relationship holds for the transfer function by the well-known Maxwell’s reciprocity theorem [25].

(2) $$G_{lm}\left(\omega \right)\;=\;G_{ml}\left(\omega \right).$$

The FRFs of the single point m when the multiple points $l\;=\;1,2,\cdots ,N_{l}$ are excited are equal to the FRFs of the multiple measurement points $l\;=\;1,2,\cdots ,N_{l}$ when the arbitrary point m is excited, as shown in Equation (3).

(3) $$\left\{\begin{array}{c} A_{1}/F_{m}\\ \vdots \\ \begin{array}{c} A_{l}/F_{m}\\ \vdots \\ A_{N}/F_{m} \end{array} \end{array}\right\}\;=\;\left\{\begin{array}{c} A_{m}/F_{1}\\ \vdots \\ \begin{array}{c} A_{m}/F_{l}\\ \vdots \\ A_{m}/F_{N} \end{array} \end{array}\right\}.$$

Here, assuming that the coupling of adjacent modes can be ignored near the frequency ($\omega \;=\;\omega_{r}$) of the r-th mode of FRFs, the FRFs have the relationship with mode shape vector $\varphi_{r}\;=\;{\left\{\Phi_{1r},\ldots ,\;\Phi_{N_{l}r}\right\}}^{T},$ as shown in Equation (4).

(4) $$\left\{\begin{array}{c} A_{m}/F_{1}\left(\omega_{r}\right)\\ \vdots \\ \begin{array}{c} A_{m}/F_{l}\left(\omega_{r}\right)\\ \vdots \\ A_{m}/F_{N}\left(\omega_{r}\right) \end{array} \end{array}\right\}\;=\;\left\{\begin{array}{c} G_{1m}\left(\omega_{r}\right)\\ \vdots \\ \begin{array}{c} G_{lm}\left(\omega_{r}\right)\\ \vdots \\ G_{Nm}\left(\omega_{r}\right) \end{array} \end{array}\right\}\;=\;-\frac{\Phi_{mr}}{i\cdot 2\zeta_{r}}\left\{\begin{array}{c} \Phi_{1r}\\ \vdots \\ \begin{array}{c} \Phi_{lr}\\ \vdots \\ \Phi_{Nr} \end{array} \end{array}\right\}.$$

Therefore, the mode shapes of the target structure can be identified by Equation (5) because it is the spatial distribution of the imaginary terms of FRFs measured at a single point when multipoint excitation is performed, as in the case of multipoint measurement.

(5) {Φ1r⋮Φlr⋮ΦNr} = {Im(G1m(ωr))⋮Im(Glm(ωr))⋮Im(GNm(ωr))}.

#### 2.4.2. Wavelength-Frequency Relation Identification Method by Multipoint Vibration and Reciprocity Theorem

The dispersion relation between wavelength and frequency is the basic information of wave propagation characteristics [22]. In the literature [20], the wavelength of the vibration mode shapes at the eigen frequency matches the wavelength of the wave propagation [5], and wave propagation characteristics were estimated by connecting the wavelengths at the discrete eigen frequencies. However, based on the solution of the wave propagation equation, the above characteristics are not limited to the eigen frequency but hold in the frequency domain above the eigen frequency. In the ground field, a wavelength identification method by FFT in the spatial direction is generally used to investigate the wave propagation characteristics of the ground by utilizing this characteristic [30,31,32].

The spatial series of the imaginary terms of FRFs used for the identification of the mode shape is used as an input. Let the spatial series of imaginary terms of FRFs at a certain frequency ω be h(ω,l) = [Im(G1m(ω)),…,Im(GNlm(ω))]. This is transformed into the spectrum related to the spatial frequency z in Equation (6) by Fourier transform.

(6) $$H\left(\omega ,z\right)\;=\;\int_{-\infty }^{\infty }h\left(\omega ,l\right)e^{-izl}dl.$$

For H(ω,s) obtained by Equation (6), the spatial frequency $z\left(\omega \right)\;=\;\hat{z}\left(\omega \right)$ that maximizes H(ω,z) at a certain frequency ω is obtained, and the wavelength $\lambda \left(\omega \right)\;=\;1/\hat{z}$ in the wave propagation at that frequency ω can be obtained. However, in the vicinity of the eigen frequency, there is a region where the wave does not propagate due to the existence of the stop band. Note that it is difficult to estimate the wavelength λ(ω) in these frequency bands.

#### 2.4.3. Group Velocity and Distance Attenuation Identification Method by SMEW Measurement

Schmerr and Song [26] derived the reciprocity theorem in wave propagation as Equation (7), based on the dynamic equilibrium state of surface force and inertial force.

(7) $$\int_{S}^{}t_{l}\left(n\right)\cdot u_{m}dS\;=\;\int_{S}^{}t_{m}\left(n\right)\cdot u_{l}dS.$$

where $t_{l}$ and $t_{m}$ represent surface force vectors whose normal direction of arbitrary points l and m is n, $u_{l}$ and $u_{m}$ represent displacement vectors of arbitrary points l and m, and $\int_{S}^{}dS$ represents surface integrals, respectively. Although Equation (7) is derived in a stationary field, it holds in a non-stationary field in a linear system. In fact, Takahashi and Nakahata [33] verified the reciprocity of waves in H-shaped steel materials. As a result, it was validated that when the hammering direction and the measurement direction match, the dispersion characteristics (wavelength, phase velocity, group velocity) of the wave do not change even if the excitation point and the measurement point are exchanged.

From the above, it is possible to handle the data by multipoint excitation (hammering) in the same way as the multipoint measurement in the identification of group velocity and distance attenuation in wave propagation. However, there are variations in the hammering force, unlike multipoint measurement. This problem is solved by frequency-by-frequency standardization using the wavelet transform of the excitation force shown below.

The wave propagation signal along the rail, excited by the impulse hammer excitation, is composed of many frequency components. The continuous wavelet transform (CWT) [34] was used to decompose this and estimate the wave propagation characteristics for each frequency. The CWT is calculated using a group of scaled and shifted wavelet functions. The wavelet coefficient of the accelerated time series response $a_{t}$ at the analyzed arbitrary point l can be expressed by Equation (8) [20,21,34].

(8) $$W_{t,a}\;=\;\sum_{t^{\prime }\;=\;0}^{T-1}a_{t\prime }\psi^{\prime }\left[\frac{\left(t^{\prime }-t\right)\Delta_{t}}{s}\right].$$

where ψ is the mother wavelet, s is the wavelet scale, T is the time series score, $t^{\prime }\;=\;0,1,\ldots ,T-1$, $\Delta_{t}$ is the time step, t is the continuous variable of translation, and ${\left\{\right\}}^{’}$ represents the complex conjugate. As the mother wavelet, the Morlet function was used here as in the literature [20,21]. The WPS is calculated by $\left|W_{t,a}^{2}\right|$.

If there are two synchronized acceleration responses with different positions, the group velocity and distance attenuation in wave propagation can be estimated by the above WPS [20]. Based on the time $t_{1}\left(\omega_{w}\right)$ and $t_{2}\left(\omega_{w}\right)$ where the peaks of the two acceleration responses occurred when focusing on a certain central frequency $\omega_{w}$ in WPS and the distance $L_{a}$ between the two acceleration measurement positions, the group velocity $V_{g}\left(\omega \right)$ at the central frequency $\omega_{w}$ is calculated by the following Equation (9).

(9) $$V_{g}\left(\omega_{w}\right)\;=\;\frac{L_{a}}{t_{1}\left(\omega_{w}\right)-t_{2}\left(\omega_{w}\right)}.$$

Based on the peak amplitudes $P_{1}\left(\omega_{w}\right)$ and $P_{2}\left(\omega_{w}\right)$ of the two acceleration responses and the distance $L_{a}$ of the two acceleration measurement positions when focusing on a certain central frequency $\omega_{w}$ in WPS, the distance attenuation of the wave per meter along the rail can be calculated as the energy decay of Equation (10) [35].

(10) $$\beta \left(\omega_{w}\right)\;=\;\frac{20{\mathrm{Log}}_{10}\left(P_{1}\left(\omega_{w}\right)/P_{2}\left(\omega_{w}\right)\right)}{L_{a}}.$$

The hammering point shown in Figure 3 can be used as a measurement point by the reciprocity theorem because the excitation force signal and the acceleration signal in this study are time-synchronized. Therefore, $V_{g}\left(\omega_{w}\right)$ and $\beta \left(\omega_{w}\right)$ can be calculated by WPSs at each hammering point. Although $V_{g}\left(\omega_{w}\right)$ and $\beta \left(\omega_{w}\right)$ are theoretically constant regardless of the hammering position, there are few examples of examining the sensitivity and variation with respect to the distance between the excitation positions. Therefore, in this study, the authors verified the variation by comparing $V_{g}\left(\omega_{w}\right)$ and $\beta \left(\omega_{w}\right),$ obtained at each excitation position.

The above is the case where the exciting force is constant. In reality, the exciting force is performed by the operator, and there is a certain variation. Such variations especially affect the estimation result of the distance attenuation of the wave in Equation (10). Therefore, in this study, the WPS was calculated by Equation (8) for the obtained excitation force and normalized by Equation (11) at each center frequency of the WPS.

(11) $$W_{t}\left(\omega \right)\;=\;\frac{W_{t,a}\left(\omega \right)}{W_{t,f}\left(\omega \right)}.$$

where $W_{t}\left(\omega \right)$ is the normalized wavelet coefficient, and $W_{t,f}\left(\omega \right)$ is the wavelet coefficient at the center frequency ω obtained by the wavelet transform of the exciting force time series. The error caused by the exciting force characteristics is eliminated using the WPS calculated from this $W_{t}\left(\omega \right)$ in the calculation of the above Equations (9) and (10). In this study, WPS calculated from $W_{t}\left(\omega \right)$ is called NWPS (normalized WPS).

## 3. Results of Measurements and Identifications

### 3.1. Acceleration and Excitation Force Measurement Results

**Figure 6:**
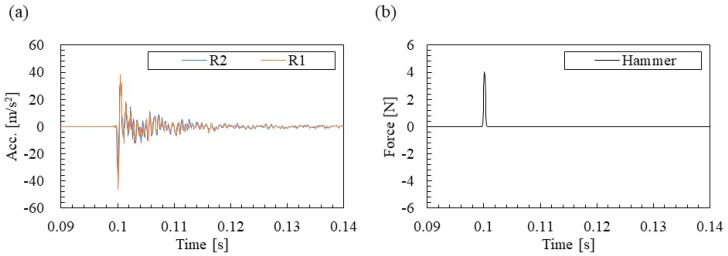
Measured time series responses: (**a**) acceleration and (**b**) excitation force.

The acceleration response and the excitation force response when the rail above the accelerometer R2 (mid-span between fastenings) is hammered are shown in Figure 6. Free vibration is excited at the rail for approximately 0.1 s when the exciting force acts. In addition, these free vibrations converge in about 0.14 s. In this study, 1 s from the time when the exciting force was maximized was used for the following analysis between the acceleration response and the exciting force response.

**Figure 7:**
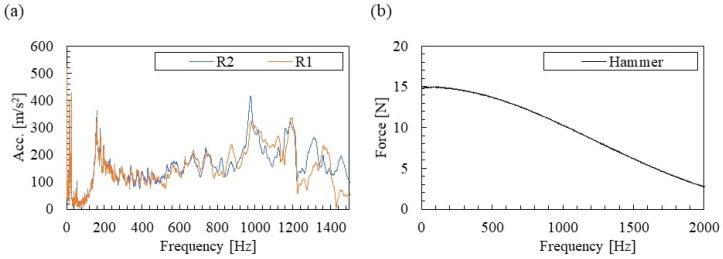
Measured spectra: (**a**) acceleration and (**b**) excitation force.

The Fourier spectrum of Figure 6 is shown in Figure 7. There are clear peaks around 160 and 980 Hz in the acceleration spectrum of Figure 7a. In addition, a peak can be confirmed at approximately 1200 Hz. The peak near 160 Hz, where the Fourier amplitudes of R1 and R2 are almost the same, is presumed to be the vertical primary mode of the rail/fastening system. In addition, the peak near 980 Hz, where the Fourier amplitude of R2 near the fastening is slightly smaller, is presumed to be the pinned–pinned mode where the fastening position is a modal node. These vibration modes are verified by the identification results of the mode shapes shown in Section 3.3. In addition, no clear peak is seen in the excitation force spectrum in Figure 7b. A low-order gain of approximately 30% is obtained up to the analysis target of about 1500 Hz, and the accuracy of the FRFs calculation is ensured without problems.

### 3.2. Eigen Frequency Identification Results

**Figure 8:**
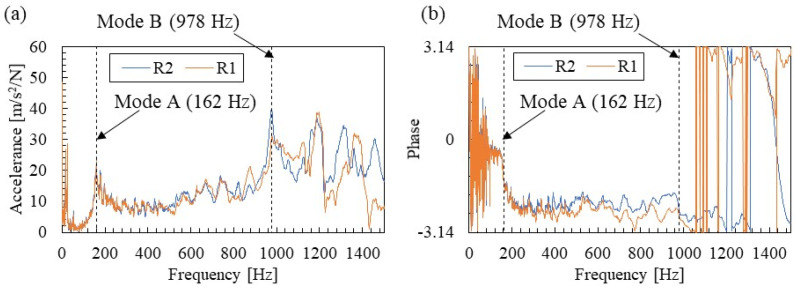
Measured FRFs: (**a**) amplitude spectrum (**b**) phase spectrum.

Figure 8 shows the amplitude and phase of FRFs obtained by dividing the acceleration spectrum of Figure 7 by the excitation force spectrum. As seen in Figure 7, the amplitude of FRFs in Figure 8a showed two main peaks, which were identified as 162 and 978 Hz, respectively. In this study, these are called Modes A and B. The phases of the FRFs in Figure 8b show a tendency for the phase to shift near the eigen frequencies of each mode, confirming that these are the eigen modes of the rail. In addition, the phase of the measurement point R2, located below the excitation point from 300 to 900 Hz, is flat. Therefore, it can be confirmed that the acceleration response and the exciting force response are time-synchronized with high accuracy. The peak observed above 1200 Hz is likely to be affected by the horizontal mode based on the literature [21]. Horizontal and torsional vibration modes are not covered in this study and will be addressed in the future.

Modes A and B can be calculated by theory. The frequency of the vertical fundamental mode (A) can be calculated by the following Equation (12) using the rail (mass) model supported by the rail pad [36].

(12) $$f_{A}\;=\;\frac{1}{2\pi }\sqrt{\frac{K_{s}}{\rho }}.$$

where $f_{A}$ is the frequency of vertical fundamental mode A, ρ is the unit length mass of the rail (60.8 kg/m), and $K_{s}$ is the rail pad spring stiffness per unit length (39.5/0.625 = 63.2 MN/m/m). The specifications of the rail are shown in Figure 3, and the specifications of the rail pad are the experimentally measured values of 39.5 MN/m converted per 1 m.

The frequency of the pinned–pinned mode (B) can be calculated by the following Equation (13) using the rail (beam) model with periodic pin supports [3].

(13) $$f_{B}\;=\;\frac{\pi }{2L^{2}}\sqrt{\frac{EI}{\rho }}\left[1-\frac{1}{2}{\left(\frac{\pi r_{g}}{L}\right)}^{2}\left(1+\frac{2\left(1+\nu \right)}{K_{s}}\right)\right].$$

where $f_{B}$ is the frequency of mode B, EI is the rail bending stiffness (6480 kNm^2^), L is the fastening interval (0.625 m), $r_{g}$ is the radius of gyration (0.063 m), ν is Poisson’s ratio, and $K_{s}$ is the shear constant of the cross-section (0.34).

Table 2 shows the identified and calculated frequencies for Models A and B. The identified frequencies are in good agreement with the frequencies calculated by the simple theoretical models above. Therefore, it can be confirmed that the modal identification result by the proposed method has certain reliability.

### 3.3. Mode Shape Identification Results

**Figure 9:**
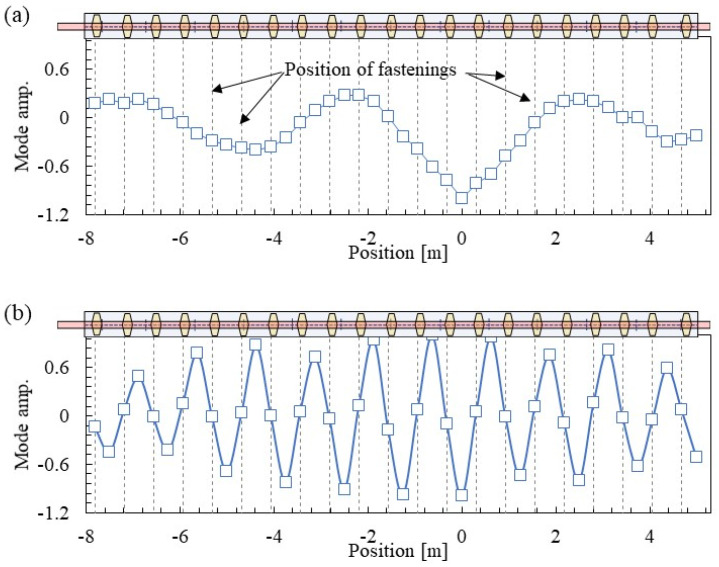
Mode shapes identified by multipoint hammering: (**a**) Mode A at 162 Hz and (**b**) Mode B at 978 Hz.

The identification results of the mode shapes calculated from all the FRFs obtained by multipoint hammering are shown in Figure 9. Here, the identification results based on the accelerometer R2 are shown. The horizontal axis shows the position where the accelerometer R2 position is 0. The position of acceleration R2 is equivalent to the vibration position in multipoint measurement by the reciprocity theorem.

The vertical bending mode with a wavelength of approximately 5.5 m centered on the accelerometer R2 is shown in Figure 9a. In addition, there is no correlation between the mode shape and the fastening position. However, in Mode B in Figure 9b, the amplitude of the mode shape is almost zero at fastening positions, and positive and negative amplitudes occur alternately at the mid-span between fastening systems. This is the well-known pinned–pinned mode.

In the past results [20,21], the spatial resolution of the mode shape was insufficient due to the limitation of the number of sensors, and numerical analysis was also used in the determination of the pinned–pinned mode. However, the proposed method of increasing the number of measurement points with a small load by multipoint excitation can clearly identify the mode shape of the pinned–pinned mode without using numerical analysis. This is a unique advantage of the proposed method in this study. It is necessary to arrange hammering points at a higher density than in this study for frequencies of 1500 Hz and above, which was the subject of this study.

### 3.4. Wavelength-Frequency Relation Identification Results

**Figure 10:**
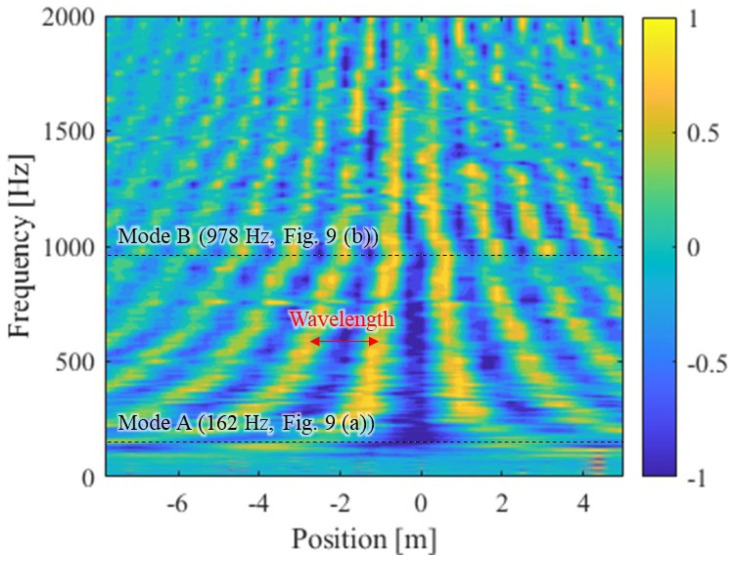
Spatial frequency distribution of imaginary terms of FRFs.

Figure 10 shows the spatial frequency distribution of the imaginary terms of FRFs for identifying the wavelength-frequency dispersion relation for wave propagation. In the figure, the vertical axis is the frequency, and the horizontal axis is the excitation position. The position of the accelerometer R2 was set to 0 on the horizontal axis. The amplitude was standardized to ±1 at each frequency.

The spatial distribution of the eigen frequencies from Modes A and B in Figure 10 is consistent with the mode shape shown in Figure 9. The region higher than the eigen frequency of Mode A and other than the frequency of each mode is the wave propagation region where wave dispersion occurs. It fluctuates spatially at regular intervals at a certain frequency, as shown in Figure 10. One cycle of this spatial fluctuation is the wavelength. It is shown that the higher the frequency, the shorter the wavelength. Clear wave propagation cannot be confirmed at frequencies lower than Mode A. This is consistent with the theory that rails behave as springs at frequencies lower than their primary vibration mode [4]. In addition, it was confirmed that the phase of the wave tends to slightly shift the low frequency of Mode B. This is the effect of the stop band, in which the wave dispersion does not occur near the eigen vibration mode of the rail [37]. By performing a Fourier transform in the spatial direction for each frequency in Figure 10, the dispersion relation between wavelength and frequency is identified.

**Figure 11:**
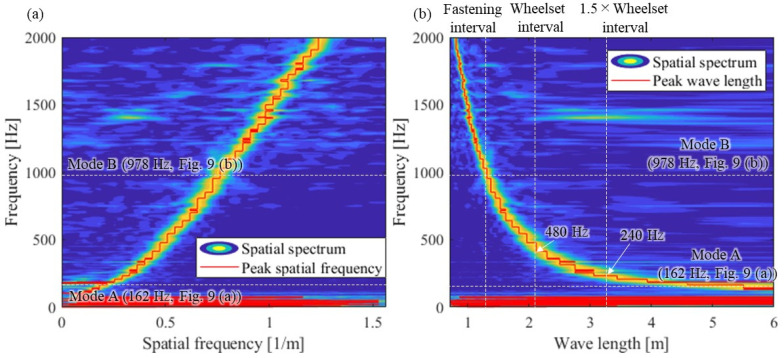
(**a**) Spatial frequency (wavenumber) frequency relation and (**b**) wavelength-frequency relation.

The spatial frequency (wavenumber) frequency relationship and the wavelength-frequency relationship are shown in Figure 11. The results of the spatial spectrum as contours to confirm the estimation accuracy of the spatial frequency are also shown in Figure 11. Figure 11 confirms that the wavelength can be estimated accurately at each frequency as the dominant component of the spatial spectrum. However, the wavelength estimation accuracy decreases at frequencies lower than Mode A because the frequency region is a region where wave propagation does not occur. The wavelength of the tested rail is approximately 6 m in Mode A (162 Hz), but the wavelength sharply shortens as the frequency increases. The wavelength of approximately 500 Hz is about 2 m. It is about 1.25 m in Mode B (978 Hz), which is the same as the length of two fastening systems. The relationships between wheelset intervals and wave propagation frequencies will be used for the discussion in Section 4.3.

From the above, it was clarified that the wavelength-frequency relationship of the actual rail, up to approximately 1200 Hz, which is pointed out to contribute to the formation mechanism of rail corrugation, can be identified by the proposed method using multipoint hammering and the reciprocity theorem. The group velocity, which shows the basic characteristics of wave propagation, can theoretically be estimated from the wavelength-frequency relationship. However, the wavelength-frequency relationship identified here is not accurate enough to calculate the slope of the curve due to the effect of spatial resolution. Additionally, distance attenuation needs to be identified separately. In the next section, the group velocity and distance attenuation of this wave are estimated by SMEW measurement.

### 3.5. SMEW Measurement Results: NWPS

**Figure 12:**
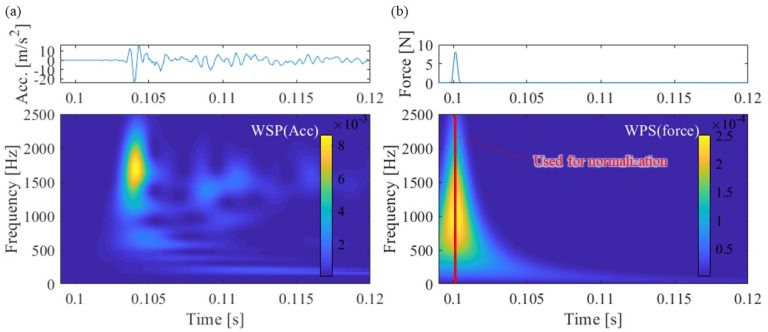
Measured responses and WPSs of the hammering at the left end of the rail: (**a**) rail R2 acceleration, (**b**) hammer excitation force.

Figure 12 shows the response of acceleration, hammering force, and WPS when hammering the leftmost excitation point shown in Figure 4a as an example of SMEW measurement.

The acceleration response occurs after 0.104 s after the excitation force acts at around 0.1 s because the hammering point is about 7.8 m away from the position of the accelerometer. The group velocity in the wave propagation of the rail is estimated based on this time difference. In addition, the distance attenuation of the wave is estimated based on the maximum amplitude that differs depending on the excitation position. However, the magnitude of WPS at each frequency of the obtained acceleration depends on the magnitude of the exciting force.

**Figure 13:**
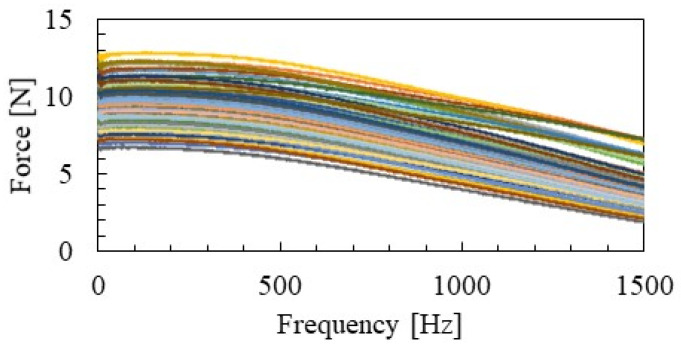
Variation in excitation force.

The spectrum variation of the hammering excitation force performed in this study is shown in Figure 13. The figure shows that the maximum value of the exciting force and the slope of the spectrum are slightly different for each hammering. To offset this difference, the WPS of acceleration was normalized by the maximum value of WPS at each frequency of the excitation force (red line in Figure 12b). This makes it possible to estimate the group velocity and distance attenuation of waves by comparing the acceleration of NWPS at different vibration positions.

**Figure 14:**
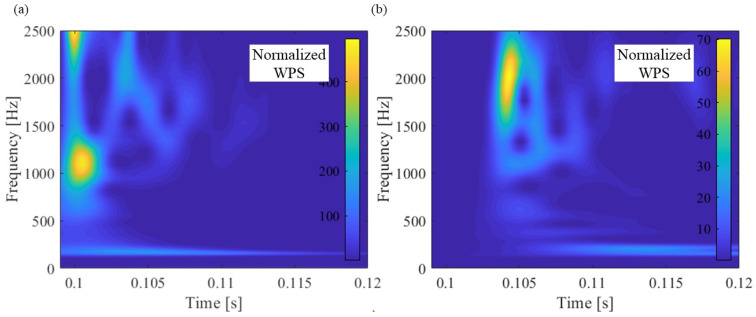
NWPS: hammering at positions (**a**) just above the R2 (point 0) and (**b**) the rail left end (point 25, 7.8 m from R2).

Figure 14 shows the NWPS of the hammering point at zero above the accelerometer R2 and hammering point 25, farthest (7.8 m) from the accelerometer R2. Hammerings were performed at approximately 0.1 s for all test cases. At the hammering point at zero, the peak of NWPS is concentrated for approximately 0.1 s when the hammering force is introduced. However, peaks occur at many frequencies of 0.03 to 0.05 s at hammering point 25. The characteristics of these wave propagations are quantified by the following group velocity frequency relations and distance attenuation frequency relations.

**Figure 15:**
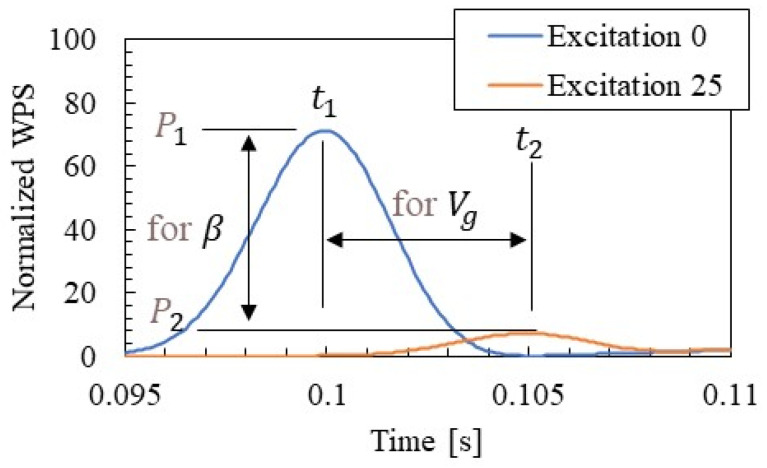
Estimating method of group velocity and distance attenuation by NWPS (506 Hz).

Figure 15 shows an example of the procedure for identifying the group velocity and distance attenuation of wave propagation by SMEW measurement as well as focusing on a certain central frequency (506 Hz) in CWT. Figure 15 shows the time series of NWPS at hammering points zero (above accelerometer R2) and 26 at 506 Hz. The group velocity is identified by the difference between the two peak time points $t_{1}\left(506\right)$ and $t_{2}\left(506\right)$ and the distance $L_{a}$ between the two points. The distance attenuation is identified by Equation (10) using the difference between the two peak amplitudes $P_{1}\left(506\right)$ and $P_{2}\left(506\right)$ and the distance $L_{a}$ between the two points.

### 3.6. SMEW Measurement Results: Group Velocity and Distance Attenuation

**Figure 16:**
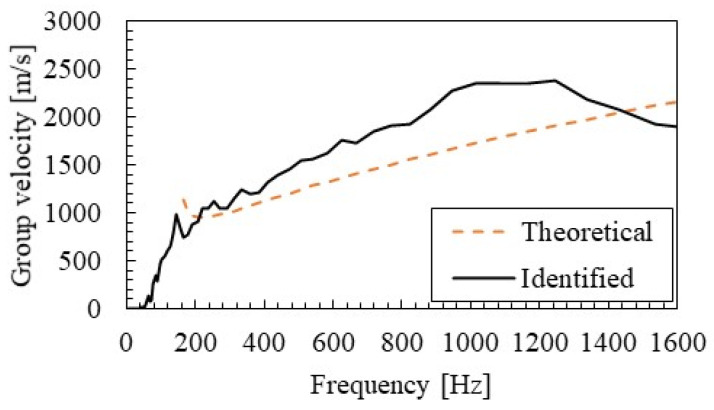
Group velocity identified by SMEW measurements and calculated by the Winkler beam theory.

Figure 16 shows the group velocities identified by SMEW measurement. The group velocity increases rapidly from around 160 Hz, which is the eigen frequency of the primary rail mode. It gradually increases up to about 1000 Hz but tends to decrease above 1200 Hz.

Figure 16 shows the group velocities obtained from the elastic supported beam (Winkler beam) theory [36,37]. The group velocity $V_{g,t}\left(\omega \right)$ of the elastic supported beam can be calculated by Equation (12).

(14) $$V_{g,t}\left(\omega \right)\;=\;\sqrt[{4}]{\frac{EI}{\rho -K_{s}/\omega^{2}}}\cdot \sqrt{\omega },\;(\omega >\sqrt{\frac{K_{s}}{\rho }}).$$

At frequencies close to the eigen frequency $\sqrt{K_{s}/\rho }$ in Mode A, the group velocity identified is about 1000 m/s and is in good agreement with the theoretical value. However, at around 1000 Hz, the identification value is up to 1.3 times larger than the theoretical value. This may be a higher-order mode such as the pinned–pinned mode, which is not considered in theory. The sophistication of the analytical and numerical models and the refinement of the parameters are the tasks of the next step.

**Figure 17:**
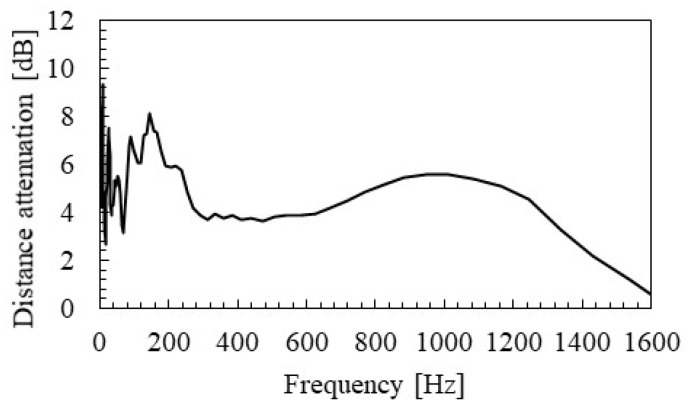
Distance attenuation identified by SMEW measurements.

Figure 17 shows the distance attenuation frequency relationship identified by SMEW measurement. It can be confirmed that the distance attenuation tends to be high, near 160 and 1000 Hz, where the eigen modes exist. Theoretically, it is known that the wave does not propagate at the frequencies of the eigen mode [38]. It is considered that this effect occurred as an increase in apparent distance attenuation. The distance attenuation is about 4 dB when focusing on the range from 300 to 600 Hz, where the influences of such eigen modes are small. This value means that the NWPS is about 25% of the hammering point at 2.1 m, which is the distance between the axles in a train bogie. In addition, when the distance exceeds 10 m, the WPS drops to about 5% of the vibration point. Therefore, it is inferred that the influence of wave interference is small at a distance greater than the distance between the axles in the bogie.

## 4. Discussions

### 4.1. Spatial Distribution of Group Velocity and Distance Attenuation

**Figure 18:**
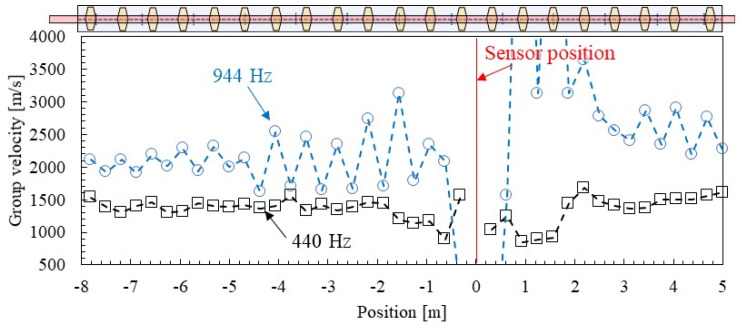
Spatial distribution of group velocities identified by SMEW measurements.

**Figure 19:**
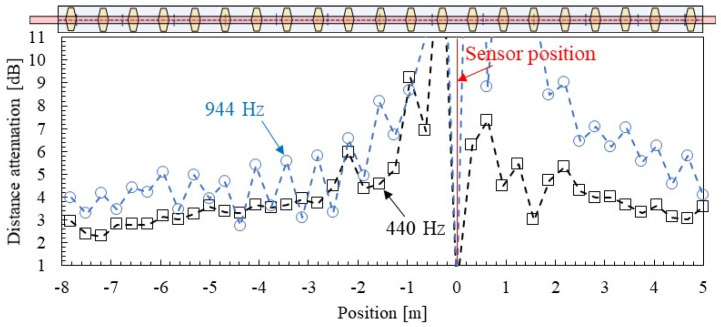
Spatial distribution of distance attenuation identified by SMEW measurements.

Figure 18 and Figure 19 show examples of the spatial distribution of group velocity and distance attenuation identified by SMEW measurements. Here, two frequencies, 440 Hz, which is different from the eigen frequency, and 944 Hz, which is near the eigen frequency, are focused.

The estimation results of each group velocity and the distance attenuation spatial distribution shown in Figure 18 and Figure 19 tend to vary greatly within a range of about 2 m from the vibration point. When the distance between the two points is short, the difference in arrival time and amplitude is small, so it is considered that a certain distance is required between the hammering point and the measurement point to obtain highly reliable results. This point will be considered in the next section based on the distribution of the error from the median value.

As for the group velocity shown in Figure 16, as the frequency approaches the eigen frequency (pinned–pinned mode), the group velocity of the wave increases, and it can be confirmed that the group velocity tends to be different between the fastening position and the mid-span of fastenings. It is suggested that the group velocity may have changed, apparently in the vicinity of the eigen frequency, where it was originally difficult for waves to propagate due to the influence of the so-called stationary mode (eigen mode).

A similar tendency can be confirmed in the distance attenuation shown in Figure 19. There is no change in the 440 Hz distance attenuation due to the fastening interval except near the measurement point with low accuracy. On the other hand, at 944 Hz, which is close to the natural frequency, the distance attenuation is higher just above the fastening than at the mid-span of fastenings. In other words, it is presumed that the influence of the mode shape has apparently occurred as the distance attenuation of the wave propagation because it is close to the pinned–pinned mode, which is stationary. Another important feature is that the distance attenuation tends to be smaller as the distance from the vibration point increases. The cause of this is presumed to be distance-dependent or amplitude-dependent [39] of attenuation (energy dissipation); however, it is unknown at this time. Therefore, when distance attenuation is evaluated as logarithmic attenuation, there may be measurement distance dependence. Compared to the position at a distance of 3 m, the attenuation at a distance of 7 m is about 75%, and the effect is not negligible. This point needs to be examined in more detail in the future.

### 4.2. Measurement Distance of Group Velocity and Distance Attenuation

**Figure 20:**
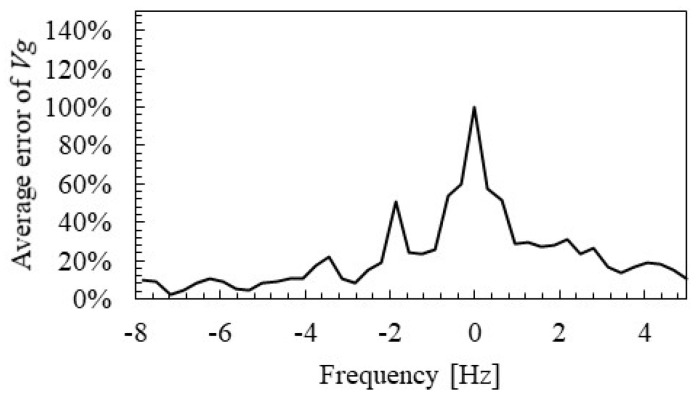
Spatial distribution of group velocity error identified by SMEW measurements.

**Figure 21:**
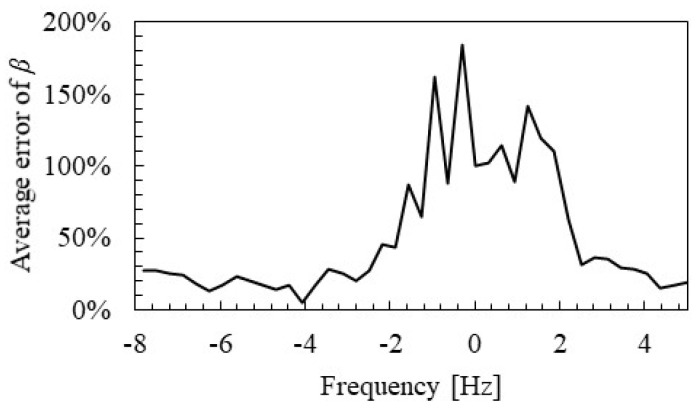
Spatial distribution of distance attenuation errors identified by SMEW measurements.

Figure 20 and Figure 21 show the spatial distribution of group velocity and distance attenuation errors identified by SMEW measurements. Based on the examination in the previous section, the error for the median value from 200 to 880 Hz, which does not overlap with the two rail-specific modes, is evaluated here. The error $E_{Vg,l}$ of the group velocity $V_{g,l}$ at the position l is calculated by the following Equation (15).

(15) $$E_{Vg,l}\;=\;\sum_{\omega =2\cdot \pi \cdot 200}^{2\cdot \pi \cdot 880}\left|\frac{V_{g,l}\left(\omega \right)-{\bar{V}}_{g}\left(\omega \right)}{{\bar{V}}_{g}\left(\omega \right)}\right|.$$

Here, ${\bar{V}}_{g}\left(\omega \right)$ is the median value of the group velocities of all measurement points. The error of distance attenuation was calculated in the same way.

Figure 20 and Figure 21 show that the estimation accuracy of group velocity and distance attenuation is ensured at approximately over 2 m. It should be noted that Figure 16 and Figure 17 are the aggregated values of only the results of 2 m or more from the measurement point based on these results.

### 4.3. Generation Mechanism of the Rail Corrugation

**Figure 22:**
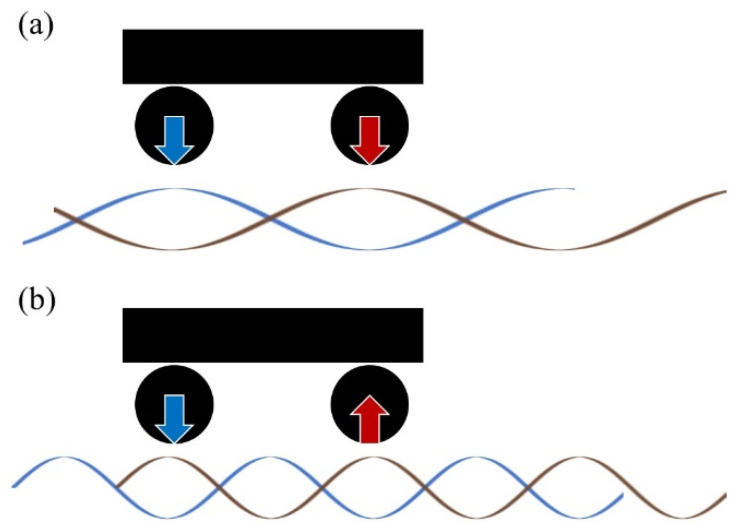
Interference of propagated waves between two wheelsets in a bogie: (**a**) an example in which waves with a wavelength twice the wheelset interval interfere in the case of in-phase wheelset excitation, (**b**) an example in which waves with a wavelength of the wheelset interval interfere in the case of antiphase wheelset excitation.

Manabe [4] and Aboshi and Tanaka [36,40] theoretically derived that the interference of propagated waves generated on the two wheelsets in a bogie, as shown in Figure 22, is one of the factors that form rail corrugation. When the contact force of two wheelsets acts on the rail, the waves generated from each other’s wheelset contact points interfere with each other. When the wave generated at one of the two wheelset contact points has an amplitude opposite to that of the contact force at the other wheelset contact point, the series impedance of the rail at the wheelset contact point becomes maximum. Since such wave interference occurs at a specific wavelength, the rail is apparently stiff with respect to the wheelset at the frequency corresponding to the wavelength of wave propagation. Therefore, if there is an initial random rail irregularity at this frequency, only the irregularity whose multiple of the wavelength matches the distance between the wheelsets in a bogie increases the contact force. Wear selectively progresses depending on such characterized contact force. Aboshi and Tanaka [36,40] theoretically clarify that such a mechanism causes rail corrugation centered on the frequency at which wave interference occurs (see [4,36,40] for details).

In this study, the wavelength-frequency relationship of the corrugated rail was identified so the above theoretical hypothesis can be verified. The distance between the wheels of the train traveling on the line is 2.1 m. The average traveling speed of the line is about 70 km/h (19.4 m/s). The wave propagation group velocity is greater than about 1000 m/s. Therefore, the Doppler effect associated with train running is less than 2%; hence, it can be almost ignored.

From the identification results of the wavelength-frequency relationship in Figure 11b, a wave with a wavelength the same as the wheelset interval of 2.1 m that interferes with the antiphase excitation of the two wheelsets corresponds to about 480 Hz. In addition, waves of 1.5 times and 0.5 times wavelengths (3.15 m and 1.05 m) that interfere with the in-phase excitation of the two wheelsets correspond to 240 and 1200 Hz, respectively. These frequencies and corresponding intervals are shown in Figure 11. Assuming that the average train traveling speed is 19.4 m/s, the vibration of 480 Hz, which causes wave interference in the antiphase, corresponds to the rail irregularity of 19.4/480 = 0.04 m. Therefore, if a periodic rail irregularity of 0.04 m (25 [1/m]) can be observed in the actual rail irregularity measurement, it can be said that the wave interference in the antiphase of the two wheelsets is the cause of the rail corrugation. Wave interferences in the in-phase (250 and 1200 Hz) correspond to the wavelengths of the rail irregularities with 0.07 m (14 [1/m]) and 0.02 m (50 [1/m]), respectively.

**Figure 23:**
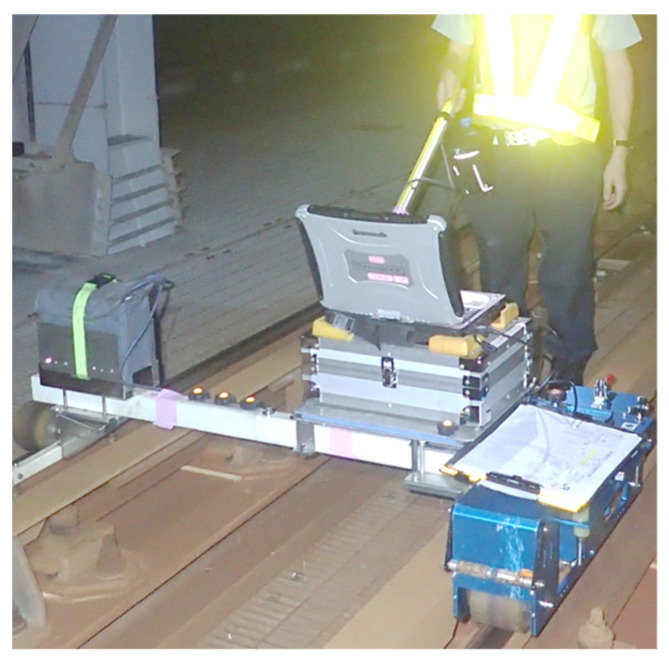
Rail irregularity continuous measuring device [41] and the measurement status.

Generally, the wavelength of rail irregularity measured by a track inspection vehicle is more than 6 m. This cannot measure the wavelengths below 0.5 m, which is the focus of this study. Therefore, the rail irregularity was measured by the other device, and the periodicity of the rail irregularity was analyzed. Figure 23 shows the rail irregularity continuous measuring device [41] used for measuring rail irregularity and the measurement status. This device can continuously measure rail irregularity, with high sensitivity in the wavelength range of 0.026 to 0.700 m.

**Figure 24:**
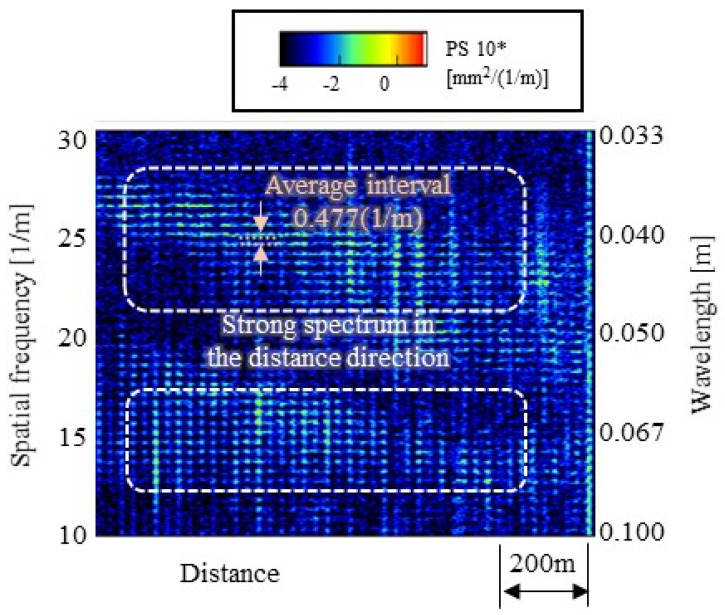
Spectrogram of the rail irregularity measured on the tested rail.

The spectrogram of the rail irregularity measured on the tested rail is shown in Figure 24. It is confirmed that a strong spectrum is continuously generated in the distance axis direction around the wavelength of 0.04 m (spatial frequency 25 [1/m]) of the irregularity. This coincides with the wavelength (of 0.04 m) calculated from the wave interference at the time of antiphase excitation of the two wheelsets. In addition, a relatively strong spectrum tends to be continuous in the distance axis direction near the wavelength of 0.07 m (14 [1/m]), though not as much as the wavelength of 0.04 m (25 [1/m]). This coincides with the wavelength calculated from the wave interference at the time of in-phase excitation of the two wheelsets based on the above. Therefore, it was confirmed that the cause of the corrugation generated on the rail was the interference of waves. In addition, in Figure 24, innumerable peaks occur at equal intervals in the spatial frequency direction. This interval is 0.477 [1/m], and its reciprocal is about 2.1 m. This is the same as the wheelset interval. Therefore, as mentioned above, it is presumed that only the rail irregularities, which are multiples of the wheelset intervals, were selectively generated around the frequency band where wave interference occurs.

It should be noted that this consideration was derived from the wave propagation characteristics of the rail alone on which the wheels are not mounted. When the train passes, there are wheels on the rails, and it is undeniable that static rail deformation and wave reflection/attenuation at the wheel positions, which are not considered here, may occur. However, Manabe [4] indicated that wave interference is one of the causes of rail corrugation, as in this study, as the result of modeling the wheels as moving masses to take this into consideration. Considering this, if the target range is limited to the wave propagation/interference that occurs between the two wheelsets in a bogie, the effect of the wheelsets is limited.

## 5. Conclusions

In this paper, a method related to rail corrugation has been developed for identifying the vertical bending vibration modes and wave propagation characteristics of rails by field tests. The proposed method can provide valuable information on operational rail modal and wave propagation characteristics entirely from field testing without the use of any numerical and computational models. The modal characteristics and wave propagation characteristics up to approximately 1500 Hz were identified by applying the proposed method to an actual rail laid on steel bridges using a direct fastening track system. The obtained results are summarized below.

- The new method developed to identify the vibration modes and wave propagation characteristics by multipoint hammering uses the reciprocity theorem to exchange the excitation and the measurement points, solving the existing method’s number-of-sensors limitation problem.
- It was empirically clarified that the pinned–pinned mode and wave propagation characteristics such as the wavelength-frequency relation up to approximately 1500 Hz can be identified only from the field tests by the high-density hammering point arrangement.
- The identified eigen frequencies were in good agreement with simple theoretical calculations.
- The SMEW measuring method that uses WPS normalized by excitation force at each frequency was proposed as a method for identifying group velocity and distance attenuation caused by multipoint hammering.
- The group velocity and distance attenuation of an actual rail identified by SMEW measurement significantly increased due to the influence of the stationary mode near the eigen modes.
- In addition, the group velocity and distance attenuation can be identified with high accuracy by the distance between the hammering point and the measurement point of over 2 m.
- From the identified wavelength-frequency relationship and the rail irregularity measurement result, the experiment confirmed that the rail corrugation with a wavelength of approximately 0.04 m was caused by the interference of the waves generated between the two wheelsets in a bogie.

The following are future considerations despite the above contributions: First, the modal damping ratio cannot be identified by the mode identification method using the FRFs adopted in this study. The modal damping ratio is an important parameter in amplitude control during resonance [42] and rail distance attenuation. It will be necessary to establish a method for identifying the mode damping ratio by a multipoint vibration test using the VAR model [43] or the ERA method [44] in the future. Following that, the cause of the non-stationarity of the distance attenuation observed in this study remains a future subject. It is necessary to construct a nonlinear mode/wave propagation identification method regarding the amplitude dependence of wave propagation characteristics. In this regard, it may be possible to elucidate the amplitude dependence of wave propagation characteristics by extending the method in the literature [45] to wave propagation. In addition, developing a numerical simulation model [20,21] that reproduces the identification results of this study is critical for mode and wave propagation control. Finally, although this study focused on vertical vibration, lateral and longitudinal vibration and wave propagation characteristics should also be clarified in the future.

## Figures and Tables

**Table 1:** List of specifications of the equipment used for measurement.

Equipment	Types	Specifications
Piezoelectric accelerometer	PV95 (Rion CO., LTD., Kokubunji-shi, Japan)	Range: 1–10,000 Hz Sensitivity: 0.714 pC/(m/s^2^)
Charge amplifier	UV16 (Rion CO., LTD., koKubunji-shi, Japan)	Range: 1–15,000 Hz
Impulse hammer	086C03 (PCB Piezotronics, New York, NY, USA)	Sensitivity: 9.68 mV/N Mass: 136 g
Recording system	NicDAQ-9189 Ni9233, Ni9239 LabVIEW (National Instruments Japan Corp., Tokyo, Japan)	Sampling frequency: 10,000 Hz

**Table 2:** Identified and calculated frequencies.

Modes	Identified Frequencies	Calculated Frequencies
A (fundamental mode)	162.0 Hz	162.2 Hz
B (pinned–pinned mode)	978.0 Hz	994.1 Hz

## Data Availability

Data sharing not applicable.
